# *Cfap97d1* is important for flagellar axoneme maintenance and male mouse fertility

**DOI:** 10.1371/journal.pgen.1008954

**Published:** 2020-08-12

**Authors:** Seiya Oura, Samina Kazi, Audrey Savolainen, Kaori Nozawa, Julio Castañeda, Zhifeng Yu, Haruhiko Miyata, Ryan M. Matzuk, Jan N. Hansen, Dagmar Wachten, Martin M. Matzuk, Renata Prunskaite-Hyyryläinen

**Affiliations:** 1 Research Institute for Microbial Diseases, Osaka University, Suita, Osaka, Japan; 2 Faculty of Biochemistry and Molecular Medicine, University of Oulu, Oulu, Finland; 3 Department of Pathology & Immunology, Baylor College of Medicine, Houston, Texas, United States of America; 4 Center for Drug Discovery, Baylor College of Medicine, Houston, Texas, United States of America; 5 Institute of Innate Immunity, Biophysical Imaging, Medical Faculty, University of Bonn, Bonn, Germany; Monash University, AUSTRALIA

## Abstract

The flagellum is essential for sperm motility and fertilization *in vivo*. The axoneme is the main component of the flagella, extending through its entire length. An axoneme is comprised of two central microtubules surrounded by nine doublets, the nexin-dynein regulatory complex, radial spokes, and dynein arms. Failure to properly assemble components of the axoneme in a sperm flagellum, leads to fertility alterations. To understand this process in detail, we have defined the function of an uncharacterized gene, *Cfap97 domain containing 1* (*Cfap97d1*). This gene is evolutionarily conserved in mammals and multiple other species, including *Chlamydomonas*. We have used two independently generated *Cfap97d1* knockout mouse models to study the gene function *in vivo*. *Cfap97d1* is exclusively expressed in testes starting from post-natal day 20 and continuing throughout adulthood. Deletion of the *Cfap97d1* gene in both mouse models leads to sperm motility defects (asthenozoospermia) and male subfertility. *In vitro* fertilization (IVF) of cumulus-intact oocytes with *Cfap97d1* deficient sperm yielded few embryos whereas IVF with *zona pellucida*-free oocytes resulted in embryo numbers comparable to that of the control. Knockout spermatozoa showed abnormal motility characterized by frequent stalling in the anti-hook position. Uniquely, *Cfap97d1* loss caused a phenotype associated with axonemal doublet heterogeneity linked with frequent loss of the fourth doublet in the sperm stored in the epididymis. This study demonstrates that *Cfap97d1* is required for sperm flagellum ultra-structure maintenance, thereby playing a critical role in sperm function and male fertility in mice.

## Author summary

Infertility is a growing issue in modern society, affecting about 15% of reproductive age couples. About 1 in 20 men of reproductive age has fertility issues. The causes of male infertility remain undefined in more than half of the cases. Approximately one-fourth of male infertility cases can be attributed to genetic factors. Currently, only a few genes involved in testis development and sperm formation have been well studied and shown to have clinical significance. A better understanding of male fertility mechanisms will help to advance infertility treatments and the development of additional contraceptive alternatives, including those targeting male sex cells. Testes uniquely express several hundreds of genes and the functions of numerous testis-specific genes are yet uncharacterized. We have identified the evolutionarily conserved *Cfap97d1* gene as a testis-specific gene in a public database screen. We used two knockout mouse models to demonstrate that the absence of the *Cfap97d1* gene causes reduced sperm motility (asthenozoospermia) due to destabilization of outer microtubule doublet in sperm flagellum. This research shows that *Cfap97d1* is an important regulator of male fertility.

## Introduction

Mammalian sperm, like most other vertebrate sperm, carry the haploid genome in the head and use the flagellum for motility [1]. The flagellum consists of the midpiece, the principal piece, and the end piece, with the axoneme extending through all three parts. In addition, the midpiece contains mitochondria, along with the outer dense fibers (ODFs) and the fibrous sheath extending to the principal piece, while the endpiece is devoid of peri-axonemal structures [1,2]. The axoneme is composed of two central singlet microtubules cylindrically surrounded by nine doublet microtubules, which is referred to as the 9 + 2 structure. The central pair of singlet microtubules are called C1 and C2. They are connected by periodic bridges and surrounded by a fibrous structure—the inner sheath, also referred to as the central pair projection (CPP) [3]. Each outer microtubule doublet (OMtD) consists of A and B tubules. The complete A tubule is fused with the incomplete B microtubule. OMtDs are associated with inner dynein arms (IDA), outer dynein arms (ODA), and the nexin-dynein regulatory complex (N-DRC) [4]. Radial spokes (RS) extend from each A tubule of the outer doublets towards the central singlets.

Sperm motility is generated by controlled sliding of OMtDs. The inner- and outer-arms of dyneins are identified as the main motor proteins, promoting sliding of microtubules along each other and resulting in flagellar bending. Bending force is generated by dyneins bound to A-tubules that are sliding on the associated B-tubules along the entire axoneme and in that way generate bending force [5]. Dynein activity is regulated by the radial spokes and central pair of microtubules [4,6]. The (a-) symmetry of the flagellar beat controls the swimming path of the sperm cell: a symmetrical flagellar beat leads to a straight swimming path of the sperm cell, whereas asymmetries in the beat pattern lead to a curved or even spiral swimming path [7,8]. Morphological or functional flagellar defects impair sperm motility (asthenozoospermia) and fertility [9,10]. Mutations in several genes have been associated with asthenozoospermia (Reviewed in [11]) including Tekt4 [12], Tecte1 [13] and others. However, the mechanism of flagellar beat regulation is not well understood.

Although various studies show that 1000–2000 genes are expressed abundantly in testis [14–16], only a fraction of these genes have been well characterized. This knowledge gap inspired our *in silico* database screens for testis-enriched genes. Wherefrom, the uncharacterized *Cfap97 domain containing 1* (*Cfap97d1*) gene emerged as a candidate. It belongs to the *cilia and flagellum associated 97* (*Cfap97*) gene family, which contains three members: *Cfap97*, *Cfap97d1*, and *Cfap97d2*. Human orthologues exist for all three members. *Cfap97* and *Cfap97d1* contain one coiled-coil domain that is absent in *Cfap97d2*. The entire protein family is poorly characterized. Human and mouse *CFAP97* is an ortholog to *Chlamydomonas reinhardtii FAP97*, which has been identified in protein extracts of demembranated axonemes [17]. A study using the proximity-dependent biotinylation assay, has identified CFAP97 in a complex with human centrosome-cilium interface proteins but did not provide further characterization [18]. *Hemingway* (*Hmw*) is suggested to be the Drosophila orthologue of *Cfap97* and *Cfap97d1*, and was shown to be required for motile cilia function in sperm flagellum and auditory sensory neurons [19]. Out of these three genes, *Cfap97d1* was exclusively expressed in testes and highly conserved among mammals, indicating its potential involvement in male fertility.

Based on these findings, we have chosen *Cfap97d1* as a candidate gene having a putative function in male fertility. To analyze gene function, we have used two *Cfap97d1* knockout mouse models, which were independently generated in two laboratories to study the function of *Cfap97d1 in vivo*. The results demonstrated that loss of the *Cfap97d1* gene in mice leads to sperm motility alterations (asthenozoospermia) associated with axoneme structural instability, and cause male fertility defects.

## Results

### *Cfap97d1* is a testis-enriched gene

**Fig 1:**
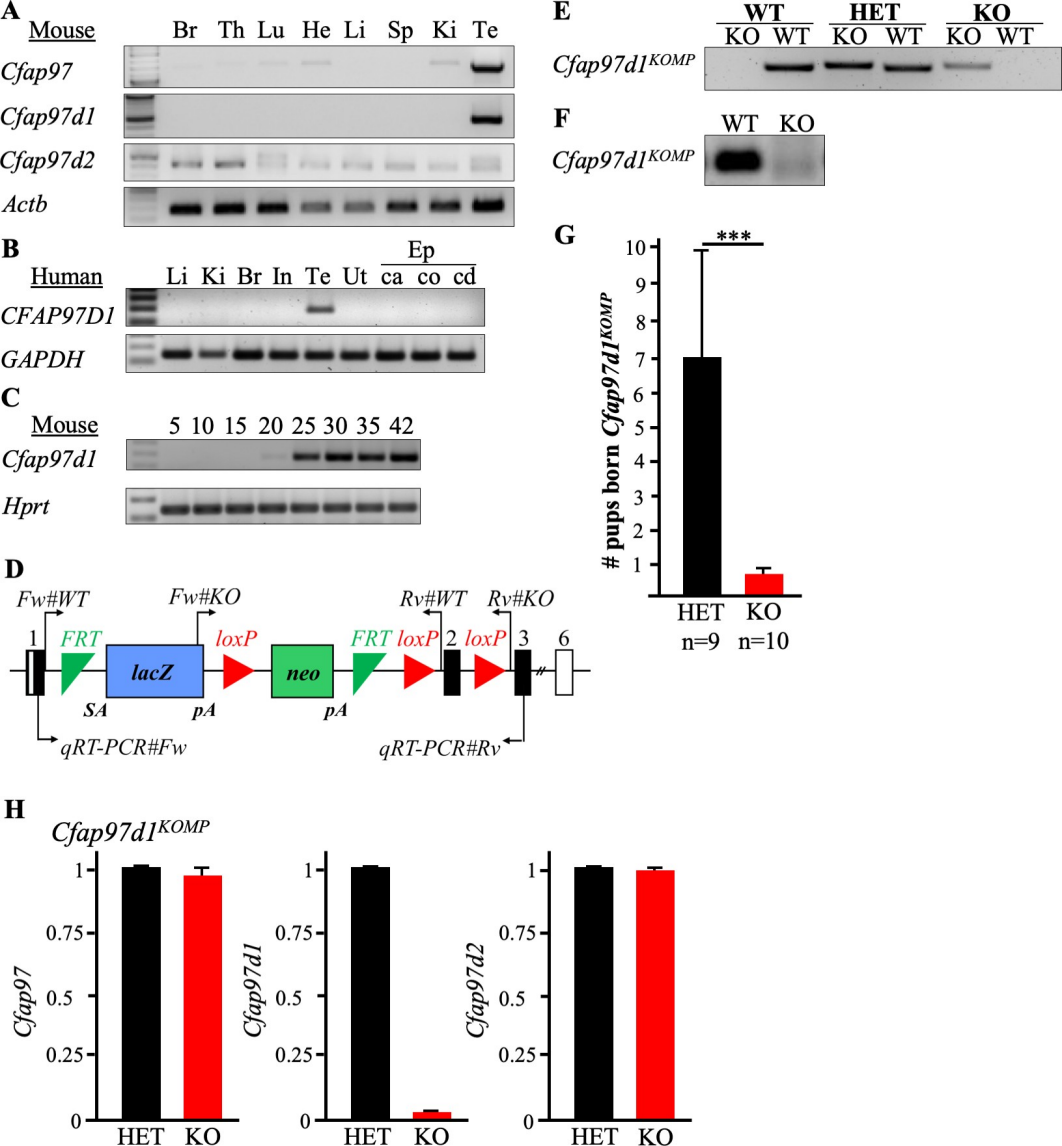
*Cfap97d1* expression in tissues, *Cfap97d1*^*KOMP*^ knockout mouse strain generation, and fertility. **(A)** Mouse multi-tissue RT-PCR profile of *Cfap97*, *Cfap97d1*, and *Cfap97d2* gene expression. *Actb* was used as a control. **(B)** Human multi-tissue RT-PCR analysis indicates that *CFAP97D1* is expressed specifically in the testes. *GAPDH* was used as a control. **(C)** *Cfap97d1* starts to be expressed in mouse testes from P20 onwards as indicated by RT-PCR analysis. *Hprt* was used as a control. **(D)** Schematic representation of the *Cfap97d1* allele generated by KOMP. Black boxes are coding alleles and white are non-coding. Genotyping primer pairs for wild type marked Fw#WT and Rv#WT, for knockout Fw#KO and Rv#KO and for qRT-PCR: qRT-PCR#Fw and qRT-PCR#Rv. **(E)** PCR genotyping of *Cfap97d1*^*KOMP*^ wild type (WT), heterozygous (HET), and knockout (KO) mice. **(F)** *Cfap97d1* deletion verification in wild type (WT), and knockout (KO) by RT-PCR. **(G)** Average number of pups born per *Cfap97d1*^*KOMPwt/-*^ and *Cfap97d1*^*KOMP-/-*^ male indicates that *Cfap97d1*^*KOMP-/-*^ males are severely sub-fertile. Error bars indicate unbiased standard deviation of number of pups born per male. **(H)** QRT-PCR expression levels of *Cfap97d*, *Cfap97d1*, and *Cfap97d2* in heterozygous control and *Cfap97d1*^*KOMP-/-*^ mice. Brain (Br), thymus (Th), lung (Lu), heart (He), liver (Li), spleen (Sp), kidney (Ki), testis (Te), intestine (In), uterus (Ut), epididymis (Ep), caput (ca), corpus (co), cauda (cd), splice acceptor (SA), polyadenylation (pA). ***P < 0.001, Student’s t-test; ±SD.

Phylogenetic analysis showed a relationship between three members of the *Cfap97* gene family: *Cfap97*, *Cfap97d1*, and *Cfap97d2* (S1A Fig). Amino acid sequence alignment demonstrated that CFAP97D1 was highly conserved among mammals (S1B Fig). To determine the actual expression profile of *Cfap97*, *Cfap97d1*, and *Cfap97d2*, we performed multi-tissue RT-PCR from adult mice. The results showed that *Cfap97d1* cDNA was detected only in mouse testes (Fig 1A, also see S1C Fig. for more tissues) whereas *Cfap97* and *Cfap97d2* were expressed in several mouse tissues (Fig 1A). Similarly, in human multi-tissue RT-PCR, *CFAP97D1* was only expressed in testes (Fig 1B). Then, we performed RT-PCR using postnatal testes of various ages to identify the time point when *Cfap97d1* gene expression starts during spermatogenesis. Data show that *Cfap97d1* expression begins around postnatal day (PND) 20, which corresponds to the late diplotene diakinesis stage and round spermatid occurrence. It is then continuously expressed from PND 25 onwards (Fig 1C).

The mouse *Cfap97d1* gene is located on chromosome 11, whereas the human orthologue *CFAP97D1* is located on chromosome 17p21. Both contain six exons, five of them are coding and one is non-coding. The mouse CFAP97D1 protein is composed of 164 amino-acid residues (S1B Fig). Based on secondary structure predictions (*i*.*e* http://www.compbio.dundee.ac.uk/) CFAP97D1 is likely to contain 4 helices (S1B Fig., grey boxes) one of which is forming a coiled-coil region (S1B Fig., blue dash box).

### *Cfap97d1* knockout male mice have severe fertility defects

To examine the function of *Cfap97d1 in vivo*, we obtained and analyzed two knockout mouse models: *Cfap97d1* (*Cfap97d1*^*tm1a(KOMP)Wtsi*^, referred to as *Cfap97d1*^*KOMP*^) mice from the Wellcome Trust Sanger Institute Knockout Mouse Project and *Cfap97d1* knockout mice generated using CRISPR/Cas9 (referred to as *Cfap97d1*^*em1*^). The cassette used to generate *Cfap97d1*^*KOMP*^ mice has FRT site followed by a *LacZ* reporter inserted between exons 1 and 2 (Fig 1D). *Cfap97d1* expression was hindered by splicing to the *LacZ* trapping element. The deletion was verified by genotyping (Fig 1E) and RT-PCR (Fig 1F) using specific primers (S1 Table). The *Cfap97d1*^*em1*^ knockout mice were generated using CRISPR/Cas9. To ensure gene disruption and to avoid an effect on the expression of the *Dusp3* gene, which is located in the vicinity of *Cfap97d1*, we designed two crRNAs targeting exon 3 and intron 5 of *Cfap97d1* (S2A Fig). We microinjected or electroporated two crRNA/tracrRNA/Cas9 ribonucleoproteins (RNPs) into zygotes [20] and obtained 20% and 33% mutants respectively (S2B Fig). The deletion of the coding region was verified by PCR (S2C Fig.; also see S2A Fig) and sequencing (S2D Fig). Knockout mice were obtained by intercrosses of heterozygous F1 with a 3168 bp deletion, referred to as *Cfap97d1*^*em1*^. Both mouse lines had similar phenotypes; the *Cfap97d1*^*KOMP*^ data is presented in the main figures of the article and *Cfap97d1*^*em1*^ data set in the Supplemental figures (S2–S6 Figs).

We did not observe gross defects in development, behavior, and survival rate in homozygous mutant mice of either strain. Next, we assessed the fertility of *Cfap97d1*^*KOMP-/-*^ and *Cfap97d1*^*em1/em1*^ knockout mice. As heterozygous males produced normal number of pups (Fig 1G and S2E Fig), we used littermate heterozygous males as controls throughout the study. The control *Cfap97d1*^*KOMPwt/-*^ males sired 7.0 ± 3.0 (SD, n = 9) pups per litter, whereas knockout males sired on average 0.6 ± 1.3 (SD, n = 10, Fig 1G). Half (50%) of *Cfap97d1*^*KOMP-/-*^ males did not sire pups at all. Similarly, the control *Cfap97d1*^*wt/em1*^ males sired 8.2 ± 2.8 (SD, n = 3) pups per litter, whereas *Cfap97d1*^*em1/em1*^ knockout males sired 0.9 ± 1.9 pups per litter (SD, n = 3, (S2E Fig)). On the other hand, we did not observe changes in female fertility. Thus, *Cfap97d1* is required for normal fertility in male mice.

Given that *Cfap97d1* knockout males occasionally sired pups, we analyzed whether this could be attributed to changed expression of other *Cfap97* family genes, namely *Cfap97* and *Cfap97d2*. We performed qRT-PCR using cDNA prepared from *Cfap97d1*^*KOMPwt/-*^ and *Cfap97d1*^*KOMP-/-*^ testes total RNA. *Cfap97d1* deletion did not affect the expression levels of *Cfap97* and *Cfap97d2* (Fig 1H) mRNA. However, this does not rule out the possibility of a change at the protein level, which could not be assessed due to lack of functional antibodies for the proteins of the *Cfap97* family.

### *Cfap97d1* knockout sperm show an impaired ability to penetrate the *zona pellucida*

**Fig 2:**
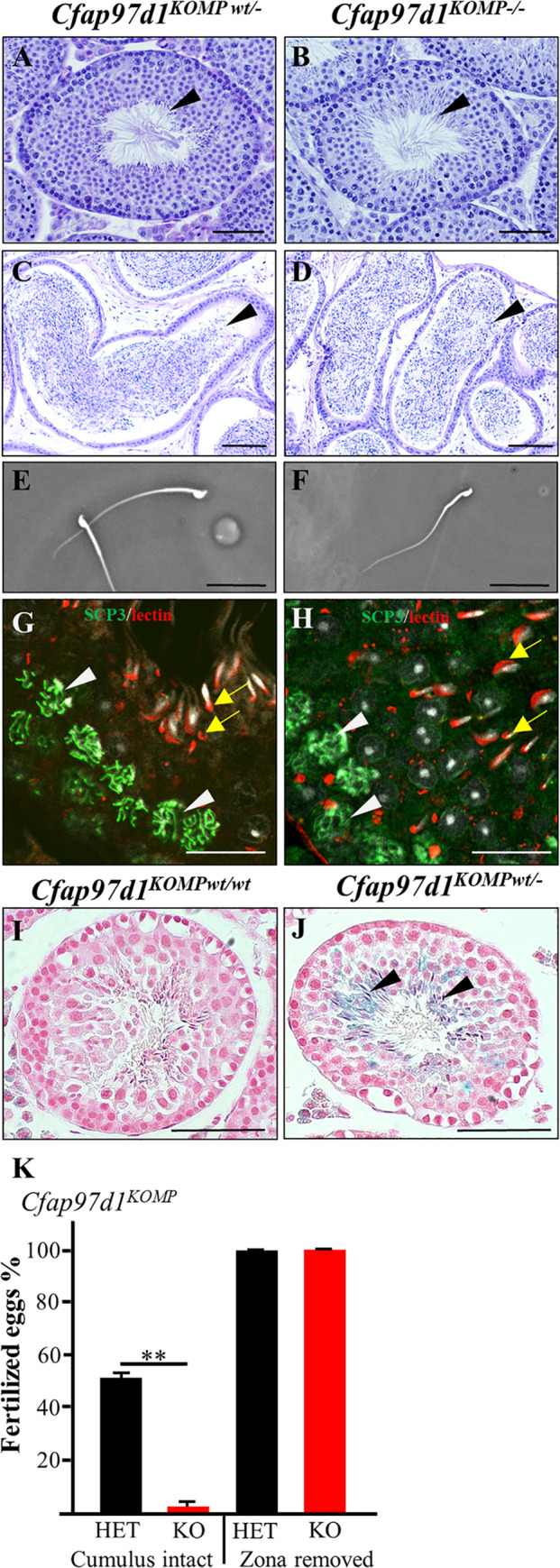
Morphology of testes and spermatozoa in *Cfap97d1* knockout, and *Cfap97d1* expression localization. **(A-D)** PAS staining of testicular sections (stage VII-VIII) from *Cfap97d1*^*KOMPwt/-*^ **(A)**, *Cfap97d1*^*KOMP-/-*^ mice 9 wks of age **(B)** and epididymis of control *Cfap97d1*^*KOMPwt/-*^ **(C)**, *Cfap97d1*^*KOMP-/-*^ mice 8 wks of age **(D)** illustrates sperm presence in the tubules (arrows). Spermatozoa collected from cauda epididymis of control *Cfap97d1*^*KOMPwt/-*^ **(E)** and *Cfap97d1*^*KOMP-/-*^ **(F)** mice do not show gross morphological changes. **(G**, **H)** Immunostainings with meiotic marker SYCP3 (green, white arrowheads) and lectin labeling acrosome (red, yellow arrows) are comparable between *Cfap97d1*^*KOMPwt/-*^ **(G)** and *Cfap97d1*^*KOMP-/-*^ **(H)** mice, nuclear staining by Hoechst (grey). **(I**, **J)** β-Galactosidase staining of wild-type **(I)** and *Cfap97d1*^*KOMPwt/-*^ testes where staining co-localizes with the elongating spermatids **(J,** black arrows). **(K)** Fertilization of intact oocytes by *Cfap97d1*^*KOMP-/-*^ sperm is significantly reduced, whereas fertilization of zona-free oocytes was comparable to control (oocytes from three mice per genotype were used for experiment). Scale bar **A-F** 50 μm; **G-J** 100 μm. Error bar indicates unbiased standard deviation of fertilization rate per male. **P < 0.01, Student’s t-test; ±SD.

Testes size (S3A–S3D Fig) and weight (S3E and S3F Fig) were not different between homozygous and control littermates of both strains. Further analysis of histological sections stained by PAS staining did not reveal any obvious defects in testes morphology in both *Cfap97d1* mouse strains (Fig 2A and 2B, S4A and S4B Fig). Epididymis cross-sections contained tubules packed with sperm in knockout *Cfap97d1*^*KOMP-/-*^ (Fig 2C and 2D) and *Cfap97d1*^*em1/em1*^ (S4C and S4D Fig) mice. Neither *Cfap97d1*^*KOMP-/-*^ (Fig 2E and 2F) nor *Cfap97d1*^*em1/em1*^ (S4E and S4F Fig) derived sperm showed obvious morphological defects.

Furthermore, meiotic progression and acrosome formation did not reveal significant differences between the *Cfap97d1*^*KOMP-/-*^ and control mice as shown by synaptonemal complex protein 3 (SYCP3), expressed during meiotic prophase, and WGA lectin, labeling acrosomes (Fig 2G and 2H). The β-galactosidase staining, that indicates *Cfap97d1* expression, was detected in elongating spermatids in *Cfap97d1*^*KOMPwt/-*^ testes (Fig 2I and 2J).

To examine sperm function, we performed an *in vitro* fertilization (IVF) assay. Both, *Cfap97d1*^*KOMP-/-*^ (Fig 2K) and *Cfap97d1*^*em1/em1*^ (S4G Fig) derived sperm displayed a reduced capability to fertilize cumulus-intact oocytes [*Cfap97d1*^*KOMP*^ line: 1.6% in knockouts and 51.2% in heterozygous controls (Fig 2K); *Cfap97d1*^*em1*^ line: 27.0% in knockouts and 88.7% in heterozygous controls (S4G Fig)]. However, the fertilization rate of *Cfap97d1* knockout spermatozoa of both strains with *zona pellucida-*free oocytes was comparable to that of the control (Fig 2K, S4G Fig). These results demonstrate that a lack of *Cfap97d1* affects sperm ability to penetrate the *zona pellucida*, resulting in severe fertility defects *in vivo*.

Finally, to determine whether the *Cfap97d1* deficient spermatozoa’s genome is intact and can contribute to the next generation, we performed intracytoplasmic sperm injection (ICSI) and IVF in zona-loosening conditions using glutathione containing medium [21,22] using the *Cfap97d1*^*em1*^ mouse line. As a result, egg activation ability of *Cfap97d1* knockout sperm heads (ICSI, (S4H Fig)) and intact sperm (zona-loosened IVF, (S4I Fig)) was similar to heterozygous controls. The number of delivered pups was comparable between the two genotypes (S4J and S4K Fig). These results demonstrate that nuclei of knockout spermatozoa have the ability to produce viable pups by using assisted reproduction techniques.

### *Cfap97d1* determines flagellar bending and frequency

**Fig 3:**
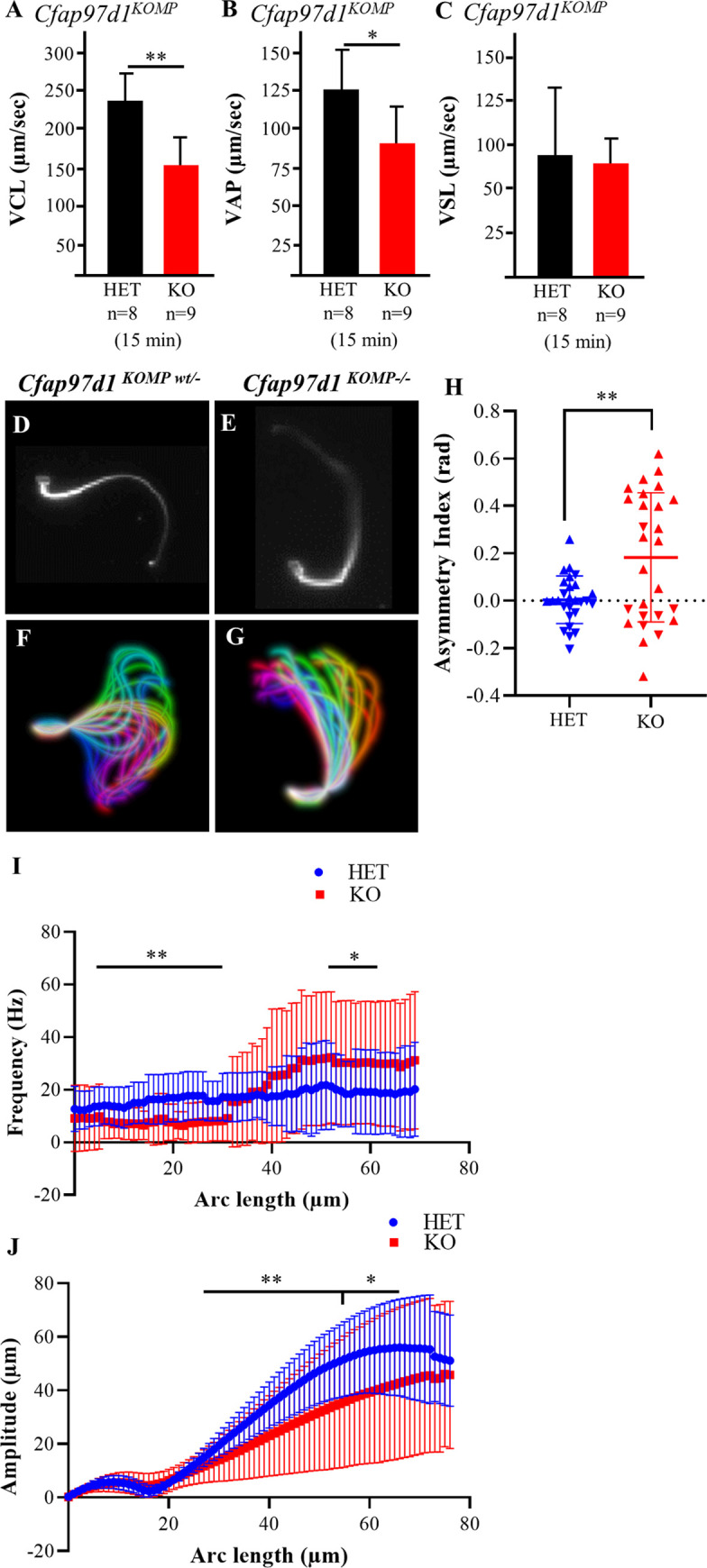
Sperm motility is altered in *Cfap97d1*^*KOMP-/-*^ mice. **(A)** Curvilinear velocity (VCL) and **(B)** average path velocity (VAP) values were reduced in *Cfap97d1*^*KOMP-/-*^ sperm. **(C)** The straight-line velocity (VSL) was not changed in *Cfap97d1*^*KOMP-/-*^ sperm. **(D**, **E)** Example of *Cfap97d1*^*KOMPwt/-*^ **(D)** and *Cfap97d1*^*KOMP-/-*^ **(E)** sperm flagellum bending modes. **(F**, **G)** Representative image of the flagellar movement waveform in tethered *Cfap97d1*^*KOMPwt/-*^ **(F)** and *Cfap97d1*^*KOMP-/-*^ **(G)** sperm. **(H)** Asymmetry index of flagellar beat in radians, determined by the median curvature angle values. Data represent the average of 0–50 μm arc length for each cell. Positive values indicate anti-hook conformation and negative values indicate pro-hook conformation. **(I)** Average primary beat frequency at each arc-length position indicates lower bending frequency of *Cfap97d1*^*KOMP-/-*^ sperm in the neck region (red line). Statistical significance occurs at 5–30 μm (**P ≤ 0.01) and 52–64 μm (*P ≤ 0.05). **(J)** Amplitude of the flagellar beat compared to head-midpiece axis in the Y- direction indicates decreased sperm flagellar moving amplitude in *Cfap97d1*^*KOMP-/-*^ (red line). Significant differences were observed from 29–56 μm (**P ≤ 0.01) and 57–67 μm, (*P ≤ 0.05). **(H-J)** Total analyzed cells = 27, from three mice of *Cfap97d1*^*KOMPwt/-*^ and *Cfap97d1*^*KOMP-/-*^. *P < 0.05, **P < 0.01, Student’s t-test, (±SD).

Next, we examined sperm motility as zona penetration defects often appear when spermatozoa show reduced motility [23,24]. The percentage of motile and progressive sperm were not significantly different between control and knockout mice of both strains (S3E and S3F Fig). We next analyzed sperm motility parameters defining the speed of sperm motion in knockout and control mice. Curvilinear velocity (VCL, the average velocity of the sperm head through its real path) and average path velocity (VAP, average velocity of the sperm head through its average trajectory) were reduced in knockout sperm compared to controls (Figs 3A, 3B, S5A and S5B), whereas straight-line velocity (VSL, average velocity of the sperm head through the straight line connecting the first position with the last track) (Fig 3C, S5C Fig) was not changed.

Next, we analyzed the flagellar beat in greater detail by recording tethered sperm tail motion at 200 frames per second (fps) with a high-speed camera (S1 and S2 Movies). The analysis revealed that the flagellar beat in *Cfap97d1*^*KOMPwt/-*^ control sperm was symmetrical with respect to a line through the midpiece (Fig 3D and 3F), whereas *Cfap97d1*^*KOMP-/-*^ sperm had a more asymmetrical waveform pattern (Fig 3E and 3G). We have quantified the difference in flagellar bending between control and *Cfap97d1*^*KOMP-/-*^ null sperm by calculating the asymmetry index. A symmetrical flagellar beat is indicated by an asymmetry index of 0. The asymmetry index of *Cfap97d1*^*KOMP-/-*^ knockout sperm was higher compared to control sperm (Fig 3H). Quantification showed that 59.3% of *Cfap97d1*^*KOMP-/-*^ sperm cells were predisposed to be in the anti-hook conformation in comparison to control 29.6% of control *Cfap97d1*^*KOMPwt/-*^ mice. The asymmetrical flagellar waveform was also recorded in *Cfap97d1*^*em1/em1*^ sperm before (S5D and S5E Fig) and after (S5F and S5G Fig) incubation (as a note: different medium and analysis technique were used than in the *Cfap97d1*^*KOMP*^ experiments presented above). Both results demonstrated that the *Cfap97d1* knockout sperm was prone to stay in the anti-hook conformation with the flagellum and the hook of the sperm head pointing in opposite directions (Fig 3H, S5E and S5G Fig). Additionally, the average flagellar beating frequency was reduced in the midpiece of *Cfap97d1*^*KOMP-/-*^ sperm at 5–30μm (**P ≤ 0.01) and 52–64 μm (*P ≤ 0.05 (Fig 3I)) as measured from the head. The amplitude of the flagellar beat with respect to the head-midpiece axis in the Y-direction was also decreased in *Cfap97d1*^*KOMP-/-*^ sperm (Fig 3J, red line, significant differences observed from 29–56 μm (**P ≤ 0.01) and 57–67 μm, (*P ≤ 0.05), total analyzed cells n = 27 from three mice of each genotype).

To analyze whether *Cfap97d1* sperm can undergo capacitation we analyzed tyrosine phosphorylation. The protein kinase A-dependent activation of tyrosine kinase results in tyrosine phosphorylation during capacitation, which can be visualized by Western blot [25,26]. The analysis did not show any difference in tyrosine phosphorylation between sperm derived from control *Cfap97d1*^*wt/em1*^ and knockout *Cfap97d1*^*em1/em1*^ mice (S6A Fig).

### *Cfap97d1* knockout sperm flagellum frequently lack microtubule doublet 4

**Fig 4:**
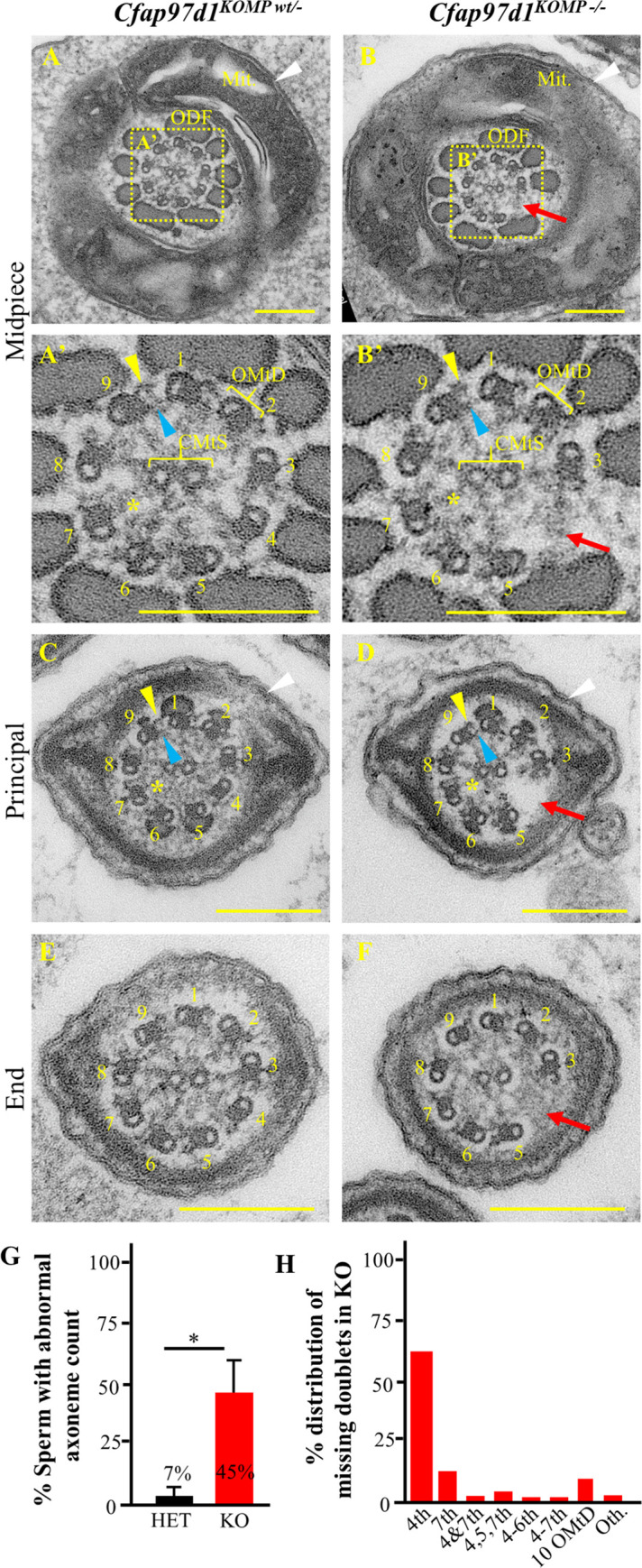
*Cfap97d1*^*KOMP-/-*^ sperm have altered doublet counts. **(A-F)** Representative transmission electron microscope micrographs (TEM). **(A**, **A’**, **C**, **E)** Normal 9+2 flagellum ultrastructure in control heterozygous sperm and **(B**, **B’**, **D**, **F)** altered axoneme counts in *Cfap97d1*^*KOMP-/-*^ mice. **(A)** Midpiece cross-section surrounded by mitochondria (Mit) and outer dense fiber (ODF) layers in control. **(B)** Micrograph depicts regular distribution of mitochondria (Mit), the rarely noted single missing one ODF and frequently missing 4^th^ outer microtubule doublet (red arrow) in *Cfap97d1*^*KOMP-/-*^ sperm. **(A’**, **B’)** Enlarged midpiece images from **A** and **B** depict outer dynein arms (yellow arrowheads), inner dynein arms (blue arrowhead), radial spokes (yellow star) and central microtubule singlets (CMtS) in control and *Cfap97d1*^*KOMP-/-*^ mouse sperm. The fourth outer microtubule doublet (OMtD) and radial spoke (yellow star) adjacent to it was frequently missing (red arrow) in *Cfap97d1*^*KOMP-/-*^ **(B’)**. **(C**, **D)** The principal piece has all intact axonemal components in control **(C)**, whereas *Cfap97d1*^*KOMP-/-*^ is missing the fourth OMtD and radial spoke adjacent to it (red arrow). **(E**, **F)** The end piece has the 9+2 structure in control **(E)** whereas *Cfap97d1*^*KOMP-/-*^ is missing the fourth OMtD (red arrow). **(G)** There were abnormal axoneme counts in 45% of *Cfap97d1*^*KOMP-/-*^ sperm and in 7% of heterozygous control sperm. **(H)** 63% of *Cfap97d1*^*KOMP-/-*^ axonemes were missing the 4^th^ OMtD. **Note**: There were 186 control and 160 *Cfap97d1*^*KOMP-/-*^ sperm cross-sections analyzed from three mice of each genotype. *P < 0.05, Student’s t-test, (±SD).

To reveal whether the defect in sperm motility in *Cfap97d1* deficient sperm is due to defects in the axoneme, we analyzed the flagellar ultrastructure of epididymal sperm of the *Cfap97d1*^*KOMP*^ line by transmission electron microscopy (TEM; Fig 4A–4F). The flagellar cross-section showed that mitochondrial sheaths in the midpiece were comparable in control and *Cfap97d1*^*KOMP-/-*^ sperm (Fig 4A and 4B). Similarly, the outer dense fiber (ODF) layer was mostly intact in controls and knockouts (Fig 4) except for the very few cases when ODF counts were not complete in *Cfap97d1*^*KOMP-/-*^ (Fig 4B). Central microtubule singlets (CMtS) were centrally located and indistinguishable in control and *Cfap97d1*^*KOMP-/-*^ mice (Fig 4A’ and 4B’). Outer microtubule doublets (OMtD) had outer- (yellow arrowhead) and inner dynein arms (blue arrowhead) attached to them as well as prominent radial spokes (yellow star in Fig 4A’ and 4B’) in control and *Cfap97d1*^*KOMP-/-*^ mice. However, the number of OMtD were irregular in 45% of flagellum in *Cfap97d1*^*KOMP-/-*^ sperm (Fig 4G) while only 7% of OMtDs were defective in control (Fig 4G). When the axoneme is viewed from the sperm head towards the tail, the outer doublets can be numbered 1 through 9 [27]. Number 1 is the doublet situated on a plane perpendicular to that bisecting the microtubules of the central pair, the next doublet clockwise is number 2 and so on (Fig 4A–4F). The following quantification using this system showed that in 63% of the cases axonemes with abnormal counts in *Cfap97d1*^*KOMP-/-*^ sperm were missing the fourth doublet (Fig 4H). The 7^th^ doublet was missing less often, in 14.8%, whereas 9.8% axonemes had 10 OMtDs. We have also quantified the cases where multiple doublets were missing but they were relatively rare (4^th^ and 7^th^ in 1.6%; 4, 5, and 7^th^ in 3.7%; 4-6^th^ in 1.23%; 4-7^th^ in 1.23%). All parts of the flagellum, mid-, principal and end pieces had missing doublets. Additionally, the radial spoke associated with the missing OMtD was also gone (Fig 4B’, 4D compare to 4A’ and 4C).

We were interested if observed abnormal OMtD counts were associated with flagellum biogenesis or OMtD destabilization. To address this question, we have done TEM analyses of the sperm flagella in testes seminiferous tubules. From these experiments, we have observed that both control and *Cfap97d1* knockout flagellum had normal (9+2) OMtD counts (S6B and S6C Fig). This indicates that *Cfap97d1* is likely not controlling the flagellum biogenesis but rather plays a role in axoneme integrity maintenance.

We next performed immunoblotting to analyze axonemal components: radial spoke protein RSPH6A, which is localized in the flagellum and associated with axoneme localization [28–31], the Dynein regulatory complex subunit 3, DRC3, which is a component of the nexin-dynein regulatory complex [6,32–34] and of Kinesin Family Member 9, KIF9, that is associated with axoneme and flagellar movement [35–38]. The analysis did not detect major differences in intensity of RSPH6A, DRC3 and KIF9 protein bands in control *Cfap97d1*^*wt/em1*^ and *Cfap97d1*^*em1/em1*^ sperm lysates (S6D Fig).

**Fig 5:**
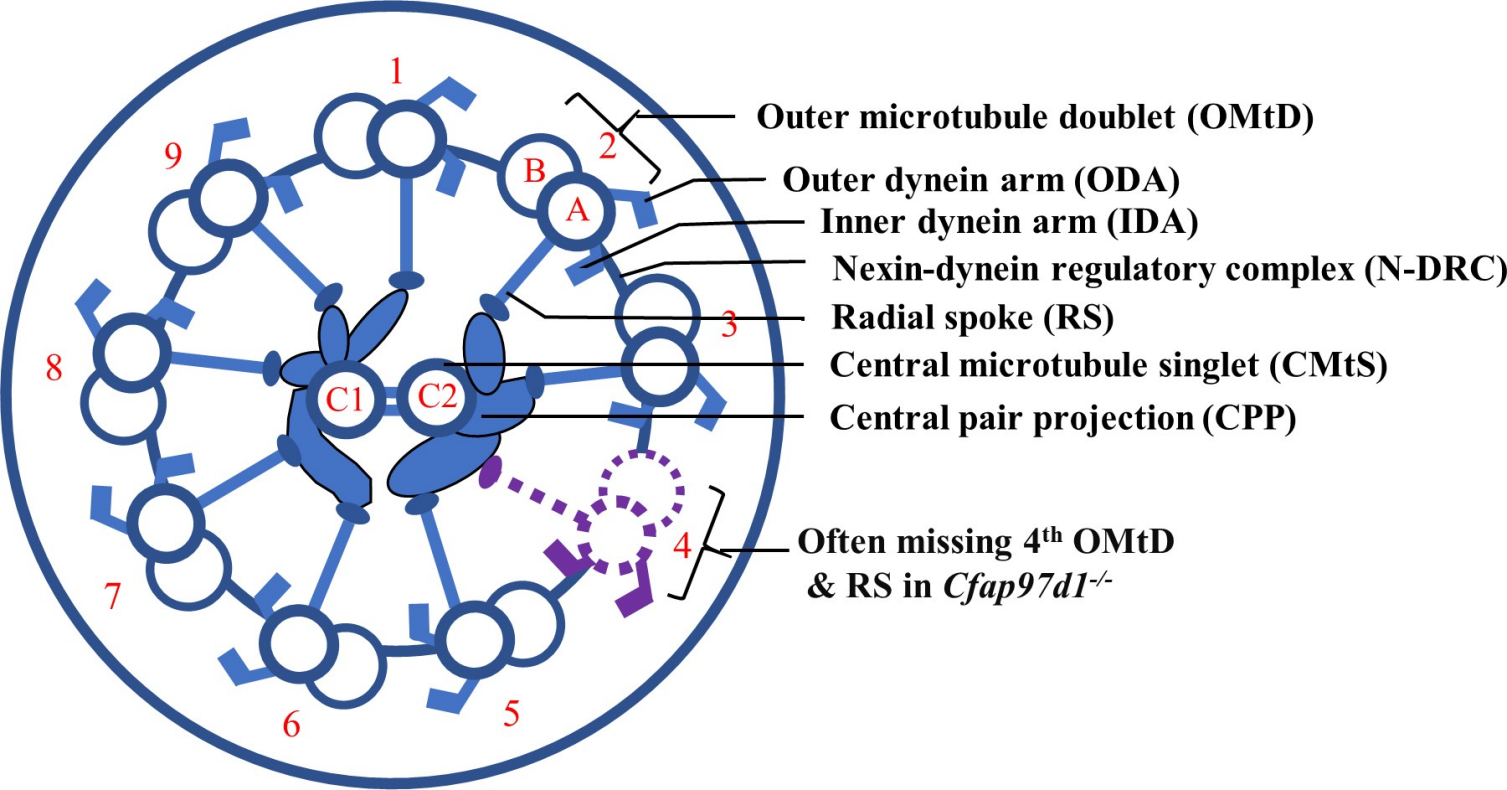
Schematic representation of the defects in axoneme caused by *Cfap97d1* deletion. Outer microtubule doublet (OMtD) composed of A and B tubules and radial spokes (RS) (blue) are labeled 1–9 (red) with the frequently missing doublet 4 and adjacent radial spoke indicated by purple dashed lines. The outer (ODA) and inner dynein arms (IDA) are attached to the A tubule of OMtD. All OMtDs are interconnected by the nexin-dynein regulatory complex (N-DRC). Central microtubule singlets (CMtS) are marked C1 and C2 with neighboring central pair projections (CPP).

Taken together our data demonstrate that *Cfap97d1* is required for axonemal doublet stabilization and sperm flagellum structural integrity (Fig 5).

## Discussion

The prerequisite for successful fertilization in mammals is sperm motility within the female reproductive tract in order to reach and fertilize the egg [39,40]. Herein, we report *Cfap97d1* as an essential gene for axoneme integrity maintenance, which is required for sperm motility and fertilization.

We have identified that *Cfap97d1* is a testis-enriched gene in humans and mice. In the mouse testes, *Cfap97d1* starts to be expressed at PND 20. This correlates with the diplotene spermatocyte stage. The expression becomes prominent from PND 25 onwards, the stage when post-meiotic round spermatids and flagella begin to form [41,42].

We have used two independent *Cfap97d1* knockout mouse models to characterize the gene function *in vivo*. About half of the *Cfap97d1* knockout male mice were infertile, while remaining males were severely sub-fertile due to asthenozoospermia, i.e. reduced sperm motility, and could sire some pups. The two independently generated mouse models presented in this article had similar sub-fertility phenotypes, excluding the possibility that sub-fertility could have been attributed to inefficient gene deletion. Even though mRNA levels of the genes from the same family (*Cfap97* and *Cfap97d2*) were not changed the possible compensatory effect on the *Cfap97d1* knockout phenotype is yet to be determined by more detailed studies. The comprehensive studies by assisted reproduction techniques (IVF and ICSI) confirmed that nuclei of *Cfap97d1* knockout spermatozoa have the ability to produce viable pups.

Sperm motility is one of the key factors determining fertility. The examination of *Cfap97d1* knockout sperm motility, by an automated CASA system, has identified a motility defect. In-depth analysis using high speed cameras has revealed that the flagellar waveform was highly asymmetric and was prone to stay in the anti-hook conformation. Correspondingly, swimming velocity was significantly reduced in mutant sperm. Additionally, flagellar beat frequency and amplitude of sperm flagella were reduced in *Cfap97d1*^*KOMP*^ knockout mice.

Flagellum formation starts in round spermatids, when the axoneme is formed, and later in elongating spermatids the accessory structures are organized. [41,42]. *Cfap97d1* is necessary for maintenance of the flagellar ultra-structure; loss of *Cfap97d1* resulted in reduced counts of outer doublets and specifically doublet 4 was frequently lost in the sperm stored in the cauda epididymis. It has been proposed that doublets 4–7 facilitate microtubule sliding during motility and doublet loss was associated with motility defects [43,44]. Failure to maintain intact doublet structure was shown to be associated with infertility, *e*.*g*. the deletion of *Ttll9* in mice caused doublet 7 shortening in the distal portion of the principal piece in sperm flagellum, along with reduction of doublet 5 polyglutamylation, leading to biased anti-hook bending and male infertility, similar to *Cfap97d1* knockout males [45]. Similarly, deletion of *Vdac3* [44], *Pla2g3* [46], and *DNAH17* [47] caused instability of sperm microtubule doublets 4–7, associated with sperm motility defects and male infertility. Additionally, in a DNAH17 missense variant, spermatozoa were disorganized during storage in cauda epididymis but not in testes, consistent with our studies. Given this, our and other laboratory studies show that doublet 4–7 stability contributes to a subtle control mechanism of microtubule sliding. Therefore, further studies will be needed to gain a thorough understanding of how this delicate system functions.

Interestingly, in *Chlamydomonas*, Pazour and colleagues have identified FAP97 (*Cfap97d1* orthologue in *Chlamydomonas reinhardtii*) in the axoneme fraction extracted by the KCl method [17]. The KCl extraction releases various axonemal proteins, including those of the inner dynein arms and the C2 central microtubule [17]. It is tempting to consider that *Cfap97d1* expression could be associated with C2 axonemal central pair apparatus proteins that are in close proximity to doublet 4 (Fig 5). However, it should be noted that our TEM analysis reveals that dynein arms and the C2 microtubule still exist in the *Cfap97d1* knockout mice. Mammalian homologs of other members of C1d or C2b have been identified. Mutations in the underlying genes, *CFAP54* (C1d) and *HYDIN* (C2b), lead to symptoms associated with primary ciliary dyskinesia including sperm motility defects [48–50].

Multiple morphological abnormalities of the sperm flagella (MMAF) is a rare syndrome that causes primary infertility. So far there are several genes associated with MMAF: CFAP43, CFAP44, CFAP69, AKAP4, DNAH1, DNAH17, CCDC39, and QRICH2 [47,51–56]. However, the genetic causes are unknown in approximately half of MMAF cases. Generally, in MMAF syndrome, sperm heads are normal and the sperm defects occur during the last stages of spermatogenesis in spermatids, when the flagellum is being assembled and undergoes elongation [57]. Thus, *Cfap97d1* could be considered as a possible candidate gene for MMAF syndrome as *Cfap97d1* deletion causes ultrastructural (but not gross-morphological) flagellar defects characteristic in MMAF. It remains to be seen how the phenotype will be affected by functional alterations of *CFAP97D1* in other species.

This study provides insight into the function of a previously uncharacterized gene and demonstrates that *Cfap97d1* is an important gene controlling structural integrity of the flagellum, sperm motility, and fertilization properties in mice.

## Materials and methods

### Transgenic animals, genotyping, and breeding

*Cfap97d1*^*tm1a(KOMP)Wtsi/+*^ (referred to as *Cfap97d1*^*KOMPwt/-*^) *LacZ*-tagged ‘knockout-first’ conditional allele mice were obtained from the KOMP consortium and maintained in a C57BL6J (C57BL/6N)/129SvEv background. The null allele in these mice is generated through splicing to a *LacZ* trapping element present in the targeting cassette [58]. Primers used for genotyping are presented in S1 Table.

The *Cfap97d1*^*wt/em1*^ mice were generated using the CRISPR-Cas9 technology (description follows) and maintained in B6D2 background.

All mice were housed in specific pathogen-free animal facilities in individually ventilated cages under light controlled conditions (12 h light/12 h dark). Animal handling was conducted in accordance with: Institutional Animal Care and Use Committees of Baylor College of Medicine, Houston, USA; Animal Care and Use Committee of the Research Institute for Microbial Diseases, Osaka University, Japan (#Biken-AP-H30-01); and Finnish Animal Ethics Committee license (38/2017), and the institutional animal care policies, which fully meet the requirements of the NIH Guide for the Care and Use of Laboratory Animals and the European Union Directive 2010/63/EU and European Convention for the protection of vertebrate animals used for experimental and other scientific purposes (ETS No. 123, appendix A). Multiple human tissues were acquired from the Human Tissue Acquisition and Pathology (HTAP) core using BCM IRB approved protocol H-14435 (Baylor College of Medicine, USA).

### Egg collection for genome editing

To prepare eggs for knockout mouse production, female mice were superovulated using injection of CARD HyperOva (0.1 mL, Kyudo, Saga, Japan) into the abdominal cavity of B6D2F1 females, followed by injection of human chorionic gonadotropin (hCG) (7.5 units, ASKA Pharmaceutical, Tokyo, Japan). Natural mating was done with B6D2F1 males 46~48 h after CARD HyperOva injection. After 19–21 h, cumulus-intact eggs were collected and treated with 0.33 mg/mL hyaluronidase (Wako, Osaka, Japan) for 5 min to remove cumulus cells for genome editing. Obtained eggs were cultured in KSOM medium [59] at 37°C under 5% CO_2_ until subsequent treatments.

### Genome editing and generation of *Cfap97d1*^*wt/em1*^ knockout mice

Pronuclear injection and electroporation were performed to introduce gRNA/Cas9 RNP as previously described [20]. Briefly, a gRNA solution was prepared by annealing crRNA (target genome sequence: 5’-AGGTGGACTGCTGGAATGAG -3' and 5’- CTTCGACTCCCACAAAGCCT -3'; Sigma-Aldrich) and tracrRNA (Sigma-Aldrich). Then, two gRNA solutions (gRNA1 and gRNA2) and Cas9 nuclease solution (Thermo Fisher Scientific) were mixed. The final concentrations of gRNA and Cas9 were as follows: for pronuclear injection, 20 ng/μL gRNA1, 20 ng/μL gRNA2, and 54 ng/μL Cas9 nuclease; for electroporation, 20 ng/μL gRNA1, 20 ng/μL gRNA2, and 100 ng/μL Cas9 nuclease.

The gRNA/Cas9 RNPs introduced embryos (B6D2F1) were transplanted into the oviduct ampulla of pseudopregnant mice (ICR; 10 embryos per ampulla) on the following day. After 19 days, pups were delivered through Caesarean section and placed with foster mothers (ICR). To generate *Cfap97d1* heterozygous mutant mice, F0 mice were mated with WT B6D2F1. Mouse colonies with a 3168 bp deletion (referred to as *Cfap97d1*^*em1*^) were maintained by sibling mating and used for the phenotype analysis. The genotyping primers are available in S1 Table. Frozen sperm from *Cfap97d1* heterozygous mutants (B6D2-*Cfap97d1* <em1Osb>, RBRC#10805, CARD#2785) are available through RIKEN BRC (http://en.brc.riken.jp/index.shtml) and CARD R-BASE (http://cardb.cc.kumamoto-u.ac.jp/transgenic/).

### RNA isolation, Reverse Transcription- and quantitative Real Time- Polymerase Chain Reaction

Mouse cDNA was prepared from multiple adult tissues of C57BL6J/129SvEv hybrid mice and testes. Briefly, tissues were dissected and snap frozen in liquid nitrogen. RNA was extracted using RNeasy Protect Mini kit (Qiagen). RNA template (0.5–1 μg/uL) was transcribed to cDNA using First Strand cDNA Synthesis Kit (Thermo Fisher Scientific) or qSCRIPT cDNA supermix (Quanta) following the manufacturer’s conditions. The generated cDNA was used to perform Reverse Transcription—(RT-PCR) and quantitative Real Time- Polymerase Chain Reaction (qRT-PCR). The primers used are listed in S1 Table. QRT-PCR was performed as described earlier [60]. Briefly, cDNA was diluted 1:10 and 1 μL was used for qRT-PCR in a total volume of 10 μL. The qRT-PCR program consisted of 40 cycles at 95°C for 30 s and at 60°C for 1 min in a CFX96 Real-Time System (BioRad) thermocycler. *Gapdh* was used for normalization by the ΔΔCT method [61].

### Histology, beta-galactosidase staining and immunostaining

*Observation of testes and sperm morphology*. Testes and epididymides were fixed in Bouin’s solution at 4°C for 8 h, followed by dehydration in increasing ethanol concentrations and embedding in paraffin. Five micrometer thick paraffin sections were hydrated in decreasing ethanol concentrations, stained with periodic acid-Schiff (PAS, Wako), counterstained with Mayer’s Hematoxylin solution (Wako), dehydrated in increasing ethanol concentrations, and finally mounted with Permount. The sections were observed using BX53 (Olympus, Tokyo, Japan) and DM LB2 (Leica, Germany) microscopes. Whole testis and cauda-epididymal sperm were observed using BX50 and BX53 microscopes with phase contrast (Olympus).

*B-galactosidase staining*. Testes were fixed overnight at 4°C (100 mM phosphate buffer, pH 7.3; 2% paraformaldehyde; 0.2% glutaraldehyde) and afterwards rinsed in wash buffer (100 mM phosphate buffer, pH 7.3; 2 mM MgCl_2_; 0.01% sodium deoxycholate; 0.02% Triton x-100) three times at room temperature. Staining was done using X-gal buffer (100 mM phosphate, pH 7.3; 2 mM MgCl_2_; 5 mM K_3_Fe(CN)_6_; 5 mM K_4_Fe(CN)_6_; 0.01% sodium deoxycholate; 0.02% Nonidet P-40; 1 mg/mL X-gal) for 12–24 h at room temperature protected from light. Samples were rinsed three times with phosphate buffered saline (PBS) for 10 min per wash. Samples were post-fixed in 10% neutral buffered formalin, dehydrated, embedded to paraffin and sectioned at 10 μm. Slides were deparaffinised, rehydrated, counterstained with Nuclear Fast Red, washed with water, and mounted with Permount.

*Immunofluorescent staining* was done as described previously [62]. Briefly, testes were dissected in PBS, fixed in 4% paraformaldehyde (PFA) overnight at 4°C, washed briefly in PBS, dehydrated through an ethanol series, embedded in paraffin, and sectioned (6 μm). After paraffin removal, rehydration antigen retrieval was done by boiling the slides in 0.01 mM citric acid buffer, pH 6, for 20 min followed by blocking in 5% fetal bovine and 5% goat serum for 1 h. The primary antibody used was SYCP3 (Abcam, ab15093, rabbit, dilution 1:100) with the secondary antibody Alexa Fluor anti-rabbit 488 (Invitrogen, A11008, dilution 1:1000). Wheat germ agglutinin, conjugated to Alexa Fluor 594 (Invitrogen, W11262, concentration 2 μg/mL) was used to stain acrosomes and Hoechst 33258 (Polyscience, Inc., concentration 5 μg/mL) was used to stain DNA. The specimens were mounted with Immu-Mount (Fisher Scientific), analyzed, and imaged by confocal microscopy (Olympus Fluoview FV10-ASW) at Biocenter Oulu Tissue Imaging Center.

### Immunoblot analysis and isolation of sperm proteins for tyrosine phosphorylation analysis

Protein lysates were resolved by SDS/PAGE under reducing condition (with 5% 2-mercaptoethanol) and transferred to PVDF membranes. After blocking with 10% skim milk, blots were incubated with primary antibodies overnight at 4°C and then incubated with secondary antibodies conjugated to horseradish peroxidase for 1 h at room temperature.

For tyrosine phosphorylation analysis spermatozoa were collected from the cauda epididymis and incubated under capacitating conditions in TYH medium [63] for 10 min. or 120 min. Spermatozoa were then collected in PBS and centrifugated at 500 x *g* for 3 min at room temperature. The collected spermatozoa were resuspended in sample buffer and boiled for 3 min. The cell debris was removed by centrifugation (15,000 x *g*, 5 min.) and the supernatant was subjected to immunoblot analysis as described above using 5% BSA instead of 10% skim milk for blocking.

Primary antibodies used: mouse anti-phosphotyrosine (1:1,000; #05–321, Sigma-Aldrich, MO, USA); mouse anti-acetylated Tubulin (1:1,000; #T7451, Sigma-Aldrich); rabbit anti-DRC3 (1:1,000; #HPA036040, Atlas Antibodies, Bromma, Sweden); goat anti-KIF9 (1:100; #sc-99958, Santa Cruz Biotechnology, CA, USA); rabbit anti-RSPH6A [30]; rabbit anti-IZUMO1 [64]. Secondary antibodies used: goat anti-rabbit IgG (1:5,000; #111-036-045; Jackson ImmunoResearch, PA, USA); goat anti-mouse IgG (1:5,000; #115-036-062; Jackson ImmunoResearch); goat anti-rat IgG (1:5,000; #112-035-167; Jackson ImmunoResearch); bovine anti-goat IgG (1:5,000; #805-035-180 Jackson ImmunoResearch).

### Fertility testing

Sexually mature *Cfap97d1*^*KOMPwt/-*^ and mutant *Cfap97d1*^*KOMP-/-*^ male mice were housed with wild-type females for at least three months, copulation was confirmed by checking for vaginal plugs and the number of pups in each cage was recorded.

### *In vitro* fertilization and genome integrity analysis by intracytoplasmic sperm injection (ICSI) and zona-loosen IVF

*In vitro* fertilization (IVF) was performed as described previously [65] with slight modifications. Briefly, cumulus-intact eggs collected from super-ovulated females 14–16 h after hCG injection (62–64 h after CARD HyperOva or PSMG injection) were placed in TYH medium [63]. To prepare zona-free eggs, eggs were treated with 1 mg/mL collagenase (Wako). Cauda sperm were collected from sexually mature males and incubated in TYH medium [63] for 2 h for capacitation. Capacitated spermatozoa were added to the drop containing eggs at a final concentration of 2 x 10^5^ sperm/mL.

ICSI was performed as previously described with some modifications [50,66]. Briefly, mature oocytes were collected from super-ovulated B6D2F1 mice. After treatment with hyaluronidase to remove cumulus cells, oocytes were placed in KSOM medium at 37°C under 5% CO_2_ until ICSI. Sperm heads were separated from tails by applying a few piezo pulses, then injected into the MII oocyte using a piezo manipulator (Prime Tech, Ibaraki, Japan).

Zona-loosened IVF was performed using CARD MEDIUM (KYUDO company, Saga, Japan) as described in the instruction manual. From vial B, 15 μL usually used for IVF with frozen-thawed sperm was used for CARD MEDIUM.

Two-cell embryos obtained by ICSI and zona-loosened IVF were transferred to pseudopregnant females the following day. Pups were genotyped at birth.

### Sperm motility analysis

Cauda sperm were extracted from control (HET) and knockout *Cfap97d1* littermates and incubated in HTF (capacitating conditions) media (Millipore) supplemented with fetal bovine serum (10%) for *Cfap97d1*^*KOMP*^ or TYH medium [63] for *Cfap97d1*^*em1*^ mice at 37°C. Sperm samples were diluted and analyzed using Hamilton Thorne’s CEROSII sperm analysis system (software version 1.5.2; Hamilton Throne Biosciences, Beverly, MA).

Motility of *Cfap97d1*^*KOMPwt/-*^ and *Cfap97d1*^*KOMP-/-*^ sperm was additionally recorded after incubation in HTF media (Millipore) supplemented with bovine serum albumin (0.3 mg/mL) using a Zeiss Axio Observer microscope mounted with a high-speed CCD camera (Hamamatsu ORCA-Flash 4.0 V2) at 200 frames per second (fps), Biocenter Oulu Tissue Imaging Center). Sperm motility was analyzed using SpermQ Analysis and Evaluator programs [67]. Briefly, the average flagellar beat frequency, amplitude, and curvature angles were calculated at each arc-length position from all frames in the recording (between 0 and 400 frames, 20.6 ± 7.4 full beat cycles). To calculate the average flagellar beat asymmetry index, the median curvature angle values were used [68]. To analyze sperm bending (pro- or anti-hook conformation), one beat cycle was observed manually to see whether the cell opened clockwise or counterclockwise into the pro-hook or anti-hook conformation. If the sperm flagellum opened clockwise into pro-hook, the median curvature angle values remained the same, but if the flagellum opened clockwise to anti-hook, the values were multiplied by -1 for normalization. A total of twenty-seven sperm cells were analyzed in capacitating conditions from three mice of each genotype, *Cfap97d1*^*KOMPwt/-*^ (n = 27 cells) and *Cfap97d1*^*KOMP-/-*^ (n = 27 cells).

Sperm motility analysis of *Cfap97d1*^*wt/em1*^ and *Cfap97d1*^*em1/em1*^ was performed as previously described [23] with slight modifications. Briefly, cauda-epididymal spermatozoa from mice used for IVF were suspended and incubated in TYH medium (capacitating condition) [63]. Sperm motility was analyzed with an Olympus BX-53 microscope equipped with a high-speed camera (HAS-L1, Ditect, Tokyo, Japan). The sperm motility was recorded at 200 frames per second. Obtained images were analyzed for beat frequencies and waveforms using sperm motion analyzing software (BohBohsoft, Tokyo, Japan).

### Transmission electron microscopy

Tissues were fixed in 1% glutaraldehyde and 4% formaldehyde in 0.1 M phosphate buffer, pH 7.4, post-fixed in 1% OsO4, dehydrated in acetone and embedded in Epon LX 112 (Ladd Research Indus- tries, VT, USA) at Biocenter Oulu Tissue Imaging Center or alternatively fixed in 2% PFA + 2.5% glutaraldehyde in 0.1 M cacodylate buffer, pH 7.4 and post-fixed in 1% OsO4 in 0.1 M cacodylate buffer + 0.2% potassium ferricyanide, following embedding in Spurr's Low Viscosity resin at Integrated Microscopy Core at Baylor College of Medicine. Semi-thin sections (1 μm) were cut and stained with toluidine blue for light microscopic inspection and selection of regions of interest. Thereafter, thin sections (80 nm) were cut and post-stained in uranyl acetate and lead citrate. Specimens were examined using the Tecnai GS Spirit microscope (FEI Europe, Edinhoven, Netherlands) and images were acquired with a Quemesa CCD camera controlled by the iTEM software (Olympus Soft Imaging Solutions GmbH, Munster, Germany) at Biocenter Oulu Tissue Imaging Center.

### Statistical analysis

Statistical analysis was performed using a two-tailed student's *t*-test (*P ≤ 0.05, **P ≤ 0.01 and ***P ≤ 0.001) by Microsoft Excel, GraphPad Prism 6 (GraphPad, San Diego, CA, USA) or R. Data represent the means ± standard deviation (±SD). At least three mice were used in each experimental group.

## Supporting information

S1 Fig Phylogenetic tree of the *CFAP97* gene family and alignment of CFAP97D1 in multiple mammalian nucleotide sequences. **(A)** Phylogenetic tree shows relation between three members of the CFAP97 family: CFAP97D1, CFAP97D2, and CFAP97. **(B)** Sequence similarity of CFAP97d1 protein is high among mammals. Red fill indicates a similarity in all species. Red letters indicate partial similarity between species. Grey boxes indicate predicted helices and the blue dashed line indicate predicted coiled-coil domain. **(C)** Mouse multi-tissue RT-PCR profile of *Cfap97d1* in multiple tissues. *Hprt* was used as a control. Heart (He), liver (Li), spleen (Sp), lung (Lu), kidney (Ki), brain (Br), stomach (St), intestine (In), testis (Te), ovary (ov), uterus (Ut).
(TIF)

S2 Fig Generation of *Cfap97d1* knockout mice strain by CRISPR/Cas9. **(A)** Gene map of *Cfap97d1*^*em1/em1*^ mouse produced using CRISPR/Cas9. Black boxes indicate coding regions, and white boxes indicate non-coding regions; black arrows: primers for genotyping, red arrows: gRNAs for genome editing. **(B)** Genome editing efficiency with gRNA/Cas9 RNPs after injection and electroporation. (**C**) Genotyping of *Cfap97d1*^*em1/em1*^ mice by PCR and deletion verification by DNA sequencing **(D)**. Four primers (Fw#1, Fw#2, Rv#1, Rv#2; also see panel A) were used for PCR. Fw#1-Rv#1 amplify the DNA sequence only from the KO allele. Fw#2-Rv#2 amplify the DNA sequence only from the WT allele, as those primers were designed inside deleted sequences. Mice with a 3168 bp deletion were used for subsequent experiments. **(E)** Number of pups born per plug detected in *Cfap97d1*^*wt/em1*^ and *Cfap97d1*^*em1/em1*^ males indicate that *Cfap97d1*^*em1/em1*^ are sub-fertile. Error bar indicates unbiased standard deviation of detected number of pups born per plug. *** P < 0.001. Student’s t-test; ±SD.
(TIF)

S3 Fig Tissue specific *Cfap97d1* expression, sub-fertility in *Cfap97d1*^*em1/em1*^ knockouts_,_ testes size, and sperm count. **(A-D)** Testes from *Cfap97d1*^*KOMPwt/-*^ **(A)**, *Cfap97d1*^*KOMP-/-*^ **(B)**, *Cfap97d1*^*wt/em1*^ **(C)**, and *Cfap97d1*^*em1/em1*^ **(D)**. Average mouse and testis weight in *Cfap97d1*^*KOMPwt/-*^ *Cfap97d1*^*KOMP-/-*^ **(E)**. Average mouse and testis weight. Motile and progressive sperm counts in *Cfap97d1*^*wt/em1*^ and *Cfap97d1*^*em1/em1*^ **(F)**. Scale bar **(A-D)** 2 mm.
(TIF)

S4 Fig Morphology of testes and spermatozoa, and *in vitro* fertilization. PAS staining of testicular sections (stage VII-VIII) of *Cfap97d1*^*wt/em1*^ **(A)** and *Cfap97d1*^*em1/em1*^ **(B).** PAS staining of epididymis sections of *Cfap97d1*^*wt/em1*^ **(C)** and *Cfap97d1*^*em1/em1*^ **(D)** illustrates sperm presence in the tubules (arrowheads). Spermatozoa collected from cauda epididymis of control *Cfap97d1*^*wt/em1*^ **(E)** and knockout *Cfap97d1*^*em1/em1*^ **(F)** does not show gross morphological changes. **(G)** IVF with cumulus-intact oocytes indicates significantly reduced fertilization ability of *Cfap97d1*^*em1/em1*^ sperm, whereas IVF with *zona pellucida*-free oocytes with *Cfap97d1*^*em1/em1*^ deficient sperm is comparable with control. Males (n = 3) each for *Cfap7d1*^*wt/em1*^ and *Cfap97d1*^*em1/em1*^ were examined (sperm concentration: 2.0x10^5^ sperm/mL). Error bar indicates unbiased standard deviation of fertilization rate per male. **(H)** The result of intracytoplasmic sperm injection (ICSI) and **(I)** *in vitr*o fertilization (IVF) under zona-loosening conditions were comparable in *Cfap7d1*^*wt/em1*^ and *Cfap97d1*^*em1/em1*^. **(J)** Pups obtained via ICSI from a homozygous male. **(K)** Genotyping of the pups obtained via (ICSI). Scale bar **A-F** 100 μm. **P < 0.01, Student’s t-test; ±SD.
(TIF)

S5 Fig Sperm motility is altered in *Cfap97d1* knockouts. (**A-C)** Sperm motility at 10 min and 120 min after sperm suspension under capacitating conditions. **(A)** VCL, curvilinear velocity and **(B)** VAP, average path velocity; were decreased in *Cfap97d1*^*em1/em1*^. **(C)** VSL, straight-line velocity was not changed in *Cfap97d1*^*em1/em1*^. (**D-G**) Flagellar bending patterns recorded after 10 min and 120 min incubation under capacitating conditions in *Cfap97d1*^*wt/em1*^ **(D**, **F)** and *Cfap97d1*^*em1/em1*^ **(E**, **G)**. Single frame throughout one beating cycle was superimposed for heterozygous and fifteen frames were superimposed for homozygous. Five spermatozoa per male (n = 3) for each condition were examined. * P < 0.05, **P < 0.01, ***P < 0.001, Student’s t-test; ±SD.
(TIF)

S6 Fig Expression of pTYR, axonemal component proteins, and 9+2 axonemal organization in sperm flagellum located in testes are not changed. **(A)** The knockout *Cfap97d1*^*em1/em1*^ (marked as *em1/em1*) Western blot analysis depicting no notable change in protein tyrosine phosphorylation (pTYR) before and after the capacitation as compared to heterozygous control *Cfap97d1*^*wt/em1*^ (*wt/em1*). The constitutively phosphorylated hexokinase band (~100 kDa) was used as a loading control. **(B**, **C)** TEM micrographs depicting undisturbed 9+2 axonemal organization in testes sperm flagellum of control and *Cfap97d1*^*KOMP-/-*^ mice. **(D)** *Cfap97d1*^*em1/em1*^ (*em1/em1)* Western blot analysis did not indicate clear differences in amount of RSPH6A, DRC3 (loading control acetylated-TUBULIN) or KIF9 (loading control IZUMO) proteins in comparison to heterozygous control *Cfap97d1*^*WT/em1*^ (*wt/em1*). Scale bar **B**, **C** 1 μm.
(TIF)

S1 Movie Tethered *Cfap97d1*^*KOMPwt/-*^ sperm cell recorded with a high speed camera at 200 fps depicting symmetric sperm flagellar movement. Movie is shown in slow motion. (AVI).
(MOV)

S2 Movie Tethered *Cfap97d1*^*KOMP-/-*^ sperm cell recorded with a high speed camera at 200 fps depicting asymmetric sperm flagellar movement. Movie is shown in slow motion (AVI).
(MOV)

S1 Table Genotyping, RT-PCR, and qRT-PCR primers. (PDF)
