# Supplementary figures and images for: Cfap97d1 is important for flagellar axoneme maintenance and male mouse fertility
Source: PLoS Genet. 2020 Aug 12;16(8):e1008954. doi: 10.1371/journal.pgen.1008954 (PMC7444823; doi:10.1371/journal.pgen.1008954)

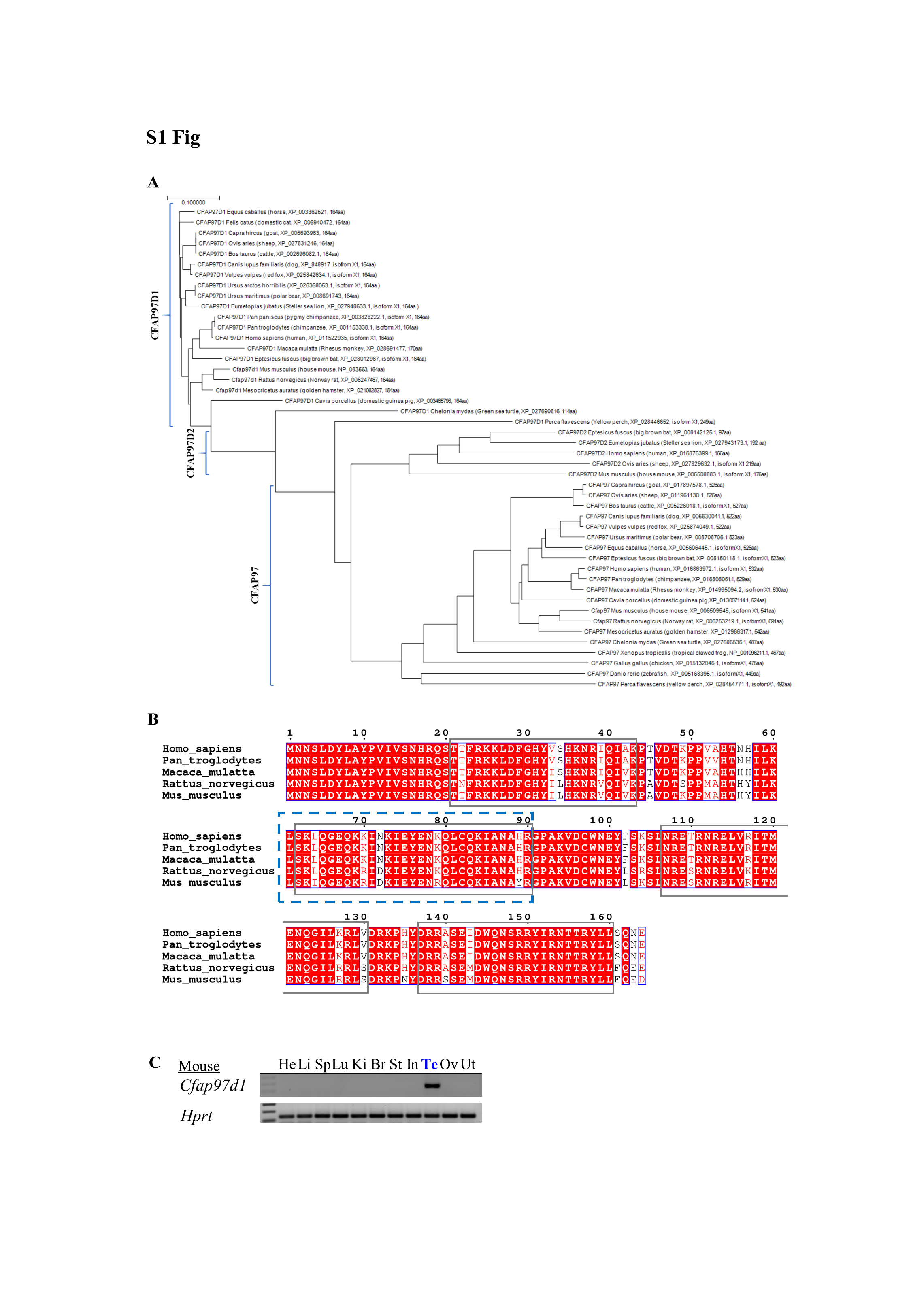

Supplement: S1 Fig — (A) Phylogenetic tree shows relation between three members of the CFAP97 family: CFAP97D1, CFAP97D2, and CFAP97. (B) Sequence similarity of CFAP97d1 protein is high among mammals. Red fill indicates a similarity in all species. Red letters indicate partial similarity between species. Grey boxes indicate predicted helices and the blue dashed line indicate predicted coiled-coil domain. (C) Mouse multi-tissue RT-PCR profile of Cfap97d1 in multiple tissues. Hprt was used as a control. Heart (He), liver (Li), spleen (Sp), lung (Lu), kidney (Ki), brain (Br), stomach (St), intestine (In), testis (Te), ovary (ov), uterus (Ut). (TIF) [file pgen.1008954.s001.TIF]

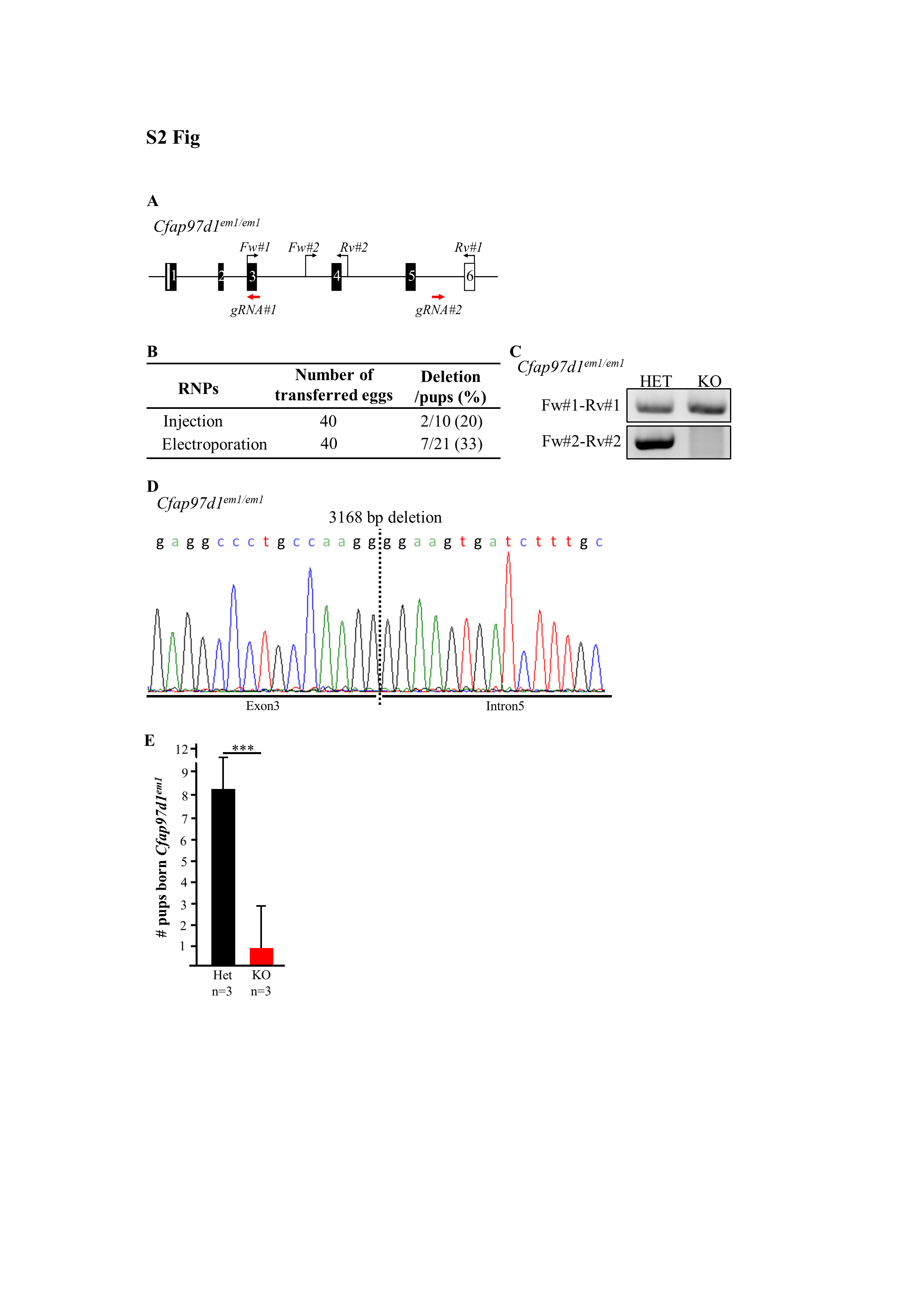

Supplement: S2 Fig — (A) Gene map of Cfap97d1em1/em1 mouse produced using CRISPR/Cas9. Black boxes indicate coding regions, and white boxes indicate non-coding regions; black arrows: primers for genotyping, red arrows: gRNAs for genome editing. (B) Genome editing efficiency with gRNA/Cas9 RNPs after injection and electroporation. (C) Genotyping of Cfap97d1em1/em1 mice by PCR and deletion verification by DNA sequencing (D). Four primers (Fw#1, Fw#2, Rv#1, Rv#2; also see panel A) were used for PCR. Fw#1-Rv#1 amplify the DNA sequence only from the KO allele. Fw#2-Rv#2 amplify the DNA sequence only from the WT allele, as those primers were designed inside deleted sequences. Mice with a 3168 bp deletion were used for subsequent experiments. (E) Number of pups born per plug detected in Cfap97d1wt/em1 and Cfap97d1em1/em1 males indicate that Cfap97d1em1/em1 are sub-fertile. Error bar indicates unbiased standard deviation of detected number of pups born per plug. *** P < 0.001. Student’s t-test; ±SD. (TIF) [file pgen.1008954.s002.TIF]

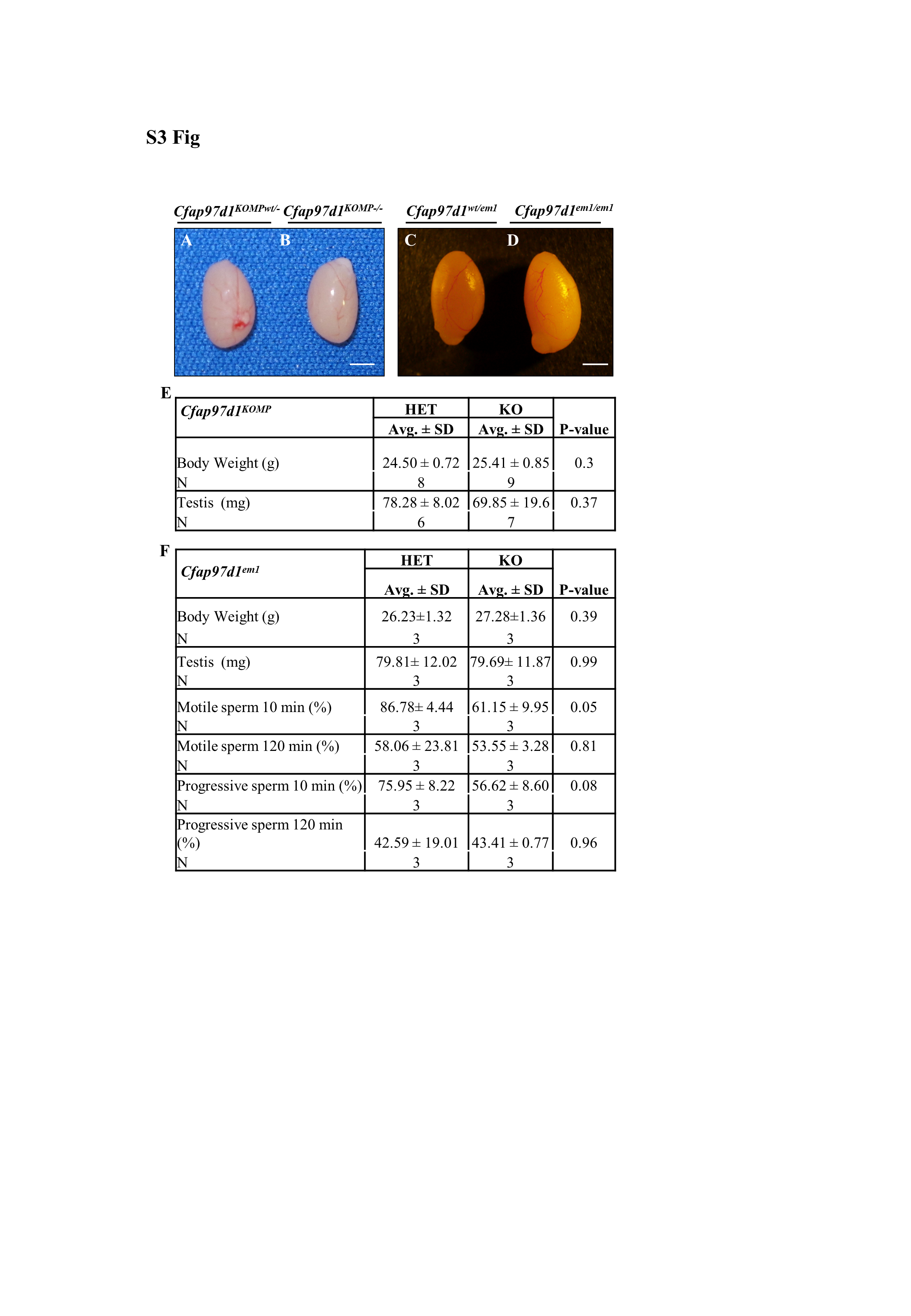

Supplement: S3 Fig — (A-D) Testes from Cfap97d1KOMPwt/- (A), Cfap97d1KOMP-/- (B), Cfap97d1wt/em1 (C), and Cfap97d1em1/em1 (D). Average mouse and testis weight in Cfap97d1KOMPwt/- Cfap97d1KOMP-/- (E). Average mouse and testis weight. Motile and progressive sperm counts in Cfap97d1wt/em1 and Cfap97d1em1/em1 (F). Scale bar (A-D) 2 mm. (TIF) [file pgen.1008954.s003.TIF]

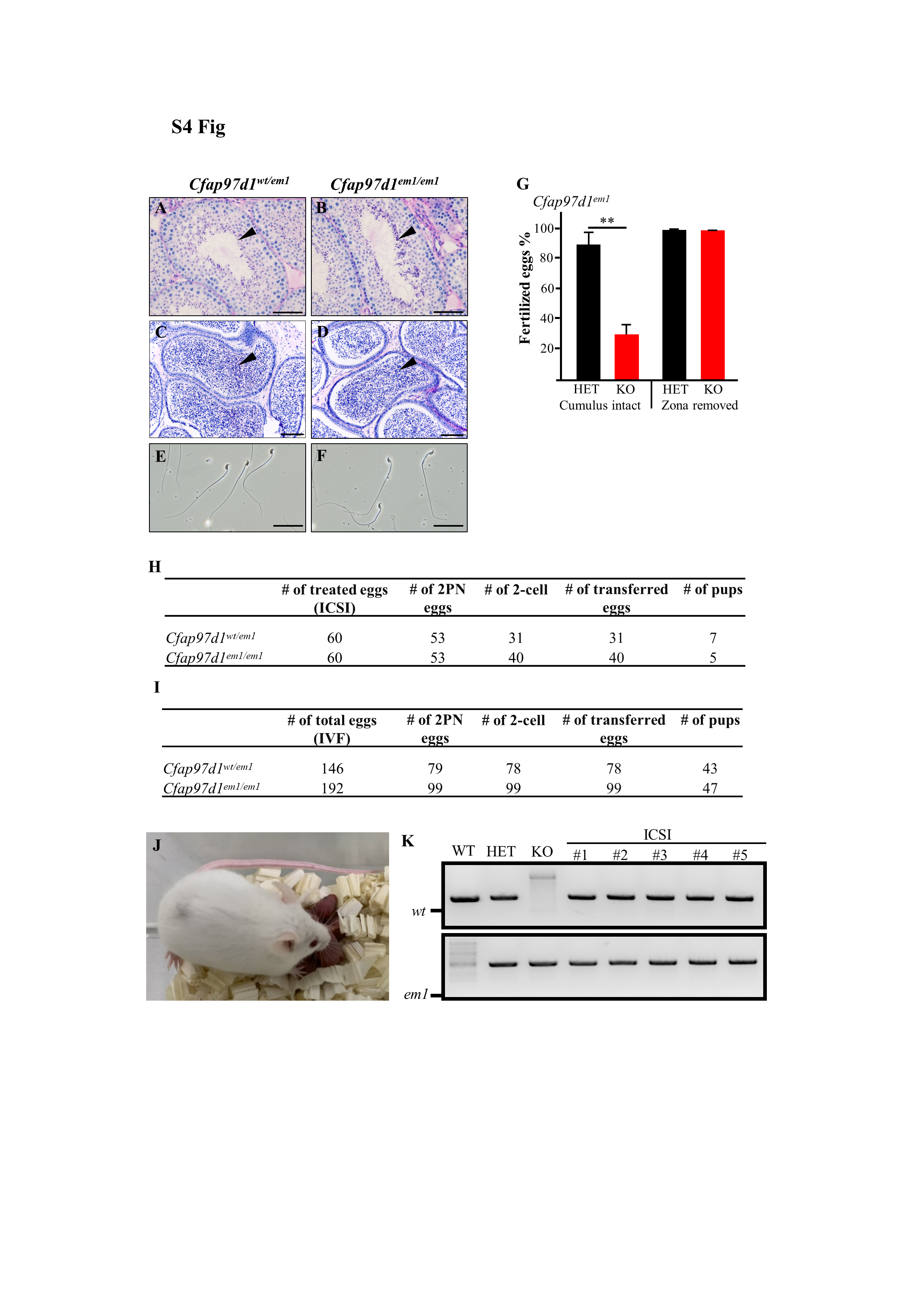

Supplement: S4 Fig — PAS staining of testicular sections (stage VII-VIII) of Cfap97d1wt/em1 (A) and Cfap97d1em1/em1 (B). PAS staining of epididymis sections of Cfap97d1wt/em1 (C) and Cfap97d1em1/em1 (D) illustrates sperm presence in the tubules (arrowheads). Spermatozoa collected from cauda epididymis of control Cfap97d1wt/em1 (E) and knockout Cfap97d1em1/em1 (F) does not show gross morphological changes. (G) IVF with cumulus-intact oocytes indicates significantly reduced fertilization ability of Cfap97d1em1/em1 sperm, whereas IVF with zona pellucida-free oocytes with Cfap97d1em1/em1 deficient sperm is comparable with control. Males (n = 3) each for Cfap7d1wt/em1 and Cfap97d1em1/em1 were examined (sperm concentration: 2.0x10^5^ sperm/mL). Error bar indicates unbiased standard deviation of fertilization rate per male. (H) The result of intracytoplasmic sperm injection (ICSI) and (I) in vitro fertilization (IVF) under zona-loosening conditions were comparable in Cfap7d1wt/em1 and Cfap97d1em1/em1. (J) Pups obtained via ICSI from a homozygous male. (K) Genotyping of the pups obtained via (ICSI). Scale bar A-F 100 μm. **P < 0.01, Student’s t-test; ±SD. (TIF) [file pgen.1008954.s004.tif]

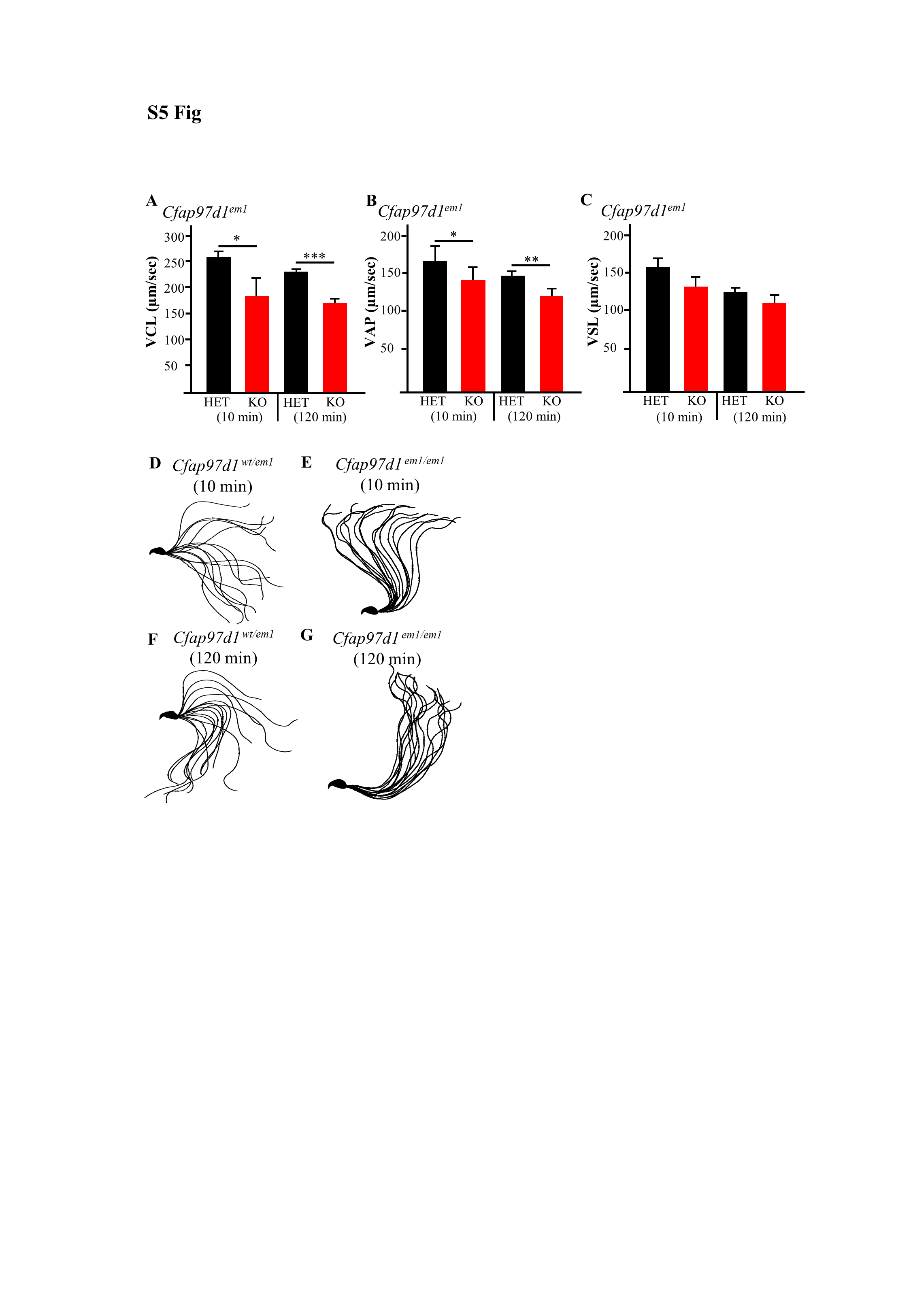

Supplement: S5 Fig — (A-C) Sperm motility at 10 min and 120 min after sperm suspension under capacitating conditions. (A) VCL, curvilinear velocity and (B) VAP, average path velocity; were decreased in Cfap97d1em1/em1. (C) VSL, straight-line velocity was not changed in Cfap97d1em1/em1. (D-G) Flagellar bending patterns recorded after 10 min and 120 min incubation under capacitating conditions in Cfap97d1wt/em1 (D, F) and Cfap97d1em1/em1 (E, G). Single frame throughout one beating cycle was superimposed for heterozygous and fifteen frames were superimposed for homozygous. Five spermatozoa per male (n = 3) for each condition were examined. * P < 0.05, **P < 0.01, ***P < 0.001, Student’s t-test; ±SD. (TIF) [file pgen.1008954.s005.TIF]

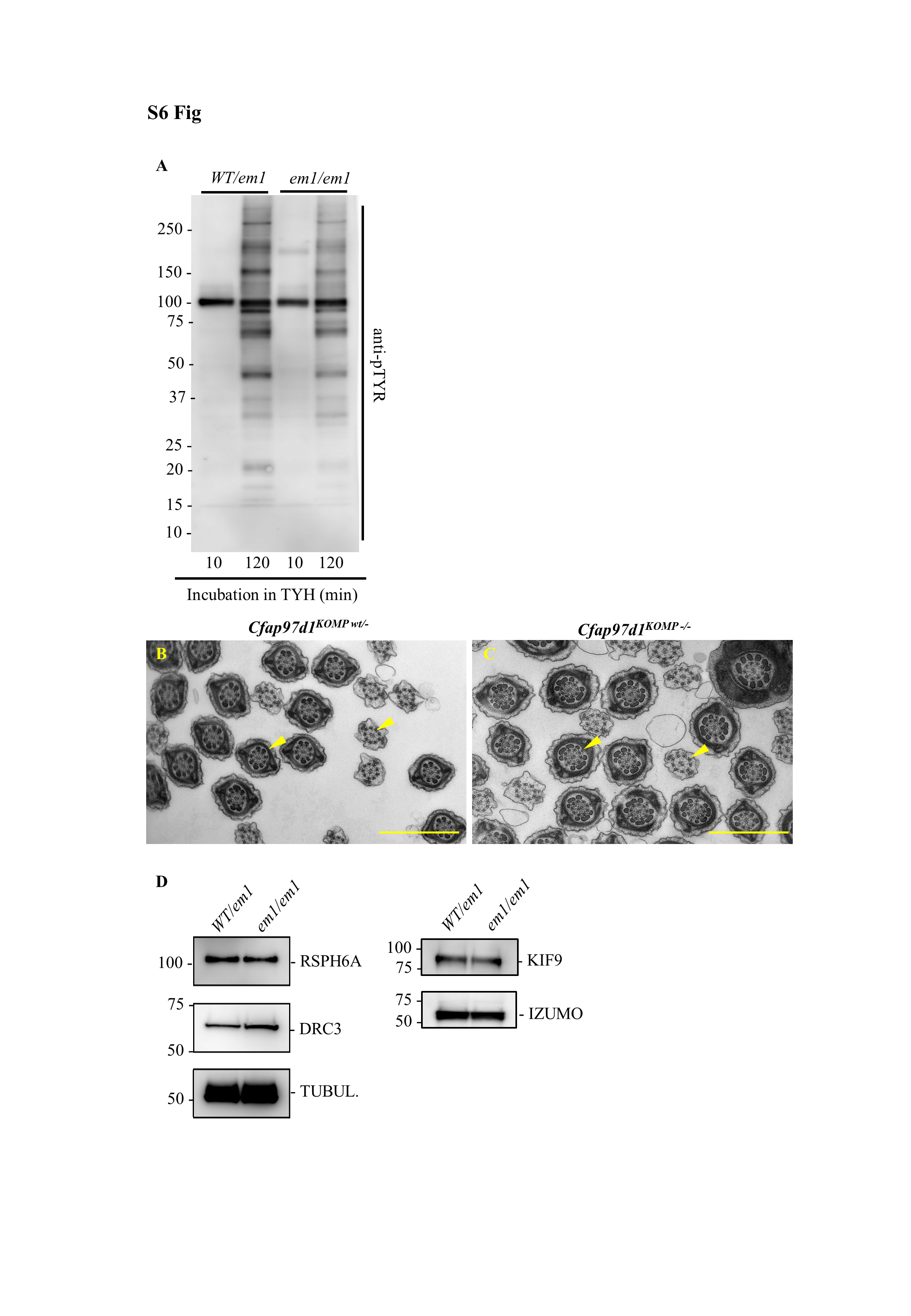

Supplement: S6 Fig — (A) The knockout Cfap97d1em1/em1 (marked as em1/em1) Western blot analysis depicting no notable change in protein tyrosine phosphorylation (pTYR) before and after the capacitation as compared to heterozygous control Cfap97d1wt/em1 (wt/em1). The constitutively phosphorylated hexokinase band (~100 kDa) was used as a loading control. (B, C) TEM micrographs depicting undisturbed 9+2 axonemal organization in testes sperm flagellum of control and Cfap97d1KOMP-/- mice. (D) Cfap97d1em1/em1 (em1/em1) Western blot analysis did not indicate clear differences in amount of RSPH6A, DRC3 (loading control acetylated-TUBULIN) or KIF9 (loading control IZUMO) proteins in comparison to heterozygous control Cfap97d1WT/em1 (wt/em1). Scale bar B, C 1 μm. (TIF) [file pgen.1008954.s006.TIF]
