# Supplementary material for: Cfap97d1 is important for flagellar axoneme maintenance and male mouse fertility
Source: PLoS Genet. 2020 Aug 12;16(8):e1008954. doi: 10.1371/journal.pgen.1008954 (PMC7444823; doi:10.1371/journal.pgen.1008954)
Supplement: S1 Table — (PDF) [file pgen.1008954.s009.pdf]

S9 Table . Genotyping, RT-PCR and qRT-PCR primers.

Gene	Forward (5’-3’)	Reverse (3’-5’)
**Genotyping**		
Cfap97d1$^{KOMPwt/wt}$	*TGGCCTTTGTCACCATTTGGA*	*TGCCATCTCTGGAGGACTCG*
Cfap97d1$^{KOMP-/-}$	*GCTACCATTACCAGTTGGTCTGGTGTC*	*AACCCCACCACAGTGACATC*
Cfap97d1$^{wt/wt}$	*ATGGTGCTGTGCGCATATCC*	*GTCAGAAAATTGCGAATGCC*
Cfap97d1$^{em1/em1}$	*GTCAGAAAATTGCGAATGCC*	*GAGATCTTAGGGTCTTCTCG*
**RT-PCR**		
hCFAP97d1	*CTGAAGTGAGACTAGGAAGAGAAG*	*GCGATTTTCTGACACAGTTGC*
hGAPDH	*AATCCCATCACCATCTTCCAG*	*ATGACCCTTTTGGCTCCC*
Cfap97	*ATGGATCGGTTTGGAGATATC*	*TTACAACCAAGCAGTACGAAC*
Cfap97d1	*ATGAACAATTCCTTGGATTA*	*CTAGTCTTCTTGGAAGAGAA*
	AACAGAGAATCAAGGAACCGAG	*CCTAAGACAGAGAATGCAGCC*
Cfap97d2	*ATGCATAGAGTCCCCCGGC*	*TCACCCCTTTGGCTTCAGGC*
Actb	*TTCTACAATGAGCTGCGTGTGGCCCC*	*GTGGTACGACCAGAGGCATACAGGGAC*
Hprt	*TGGATATGCCCTTGACTATAATGAG*	*TGGCAACATCAACAGGACTC*
**qRT-PCR**		
Cfap97	*TGACCGGGAAAATCAGAGGC*	*GATGGCCGATCGATCTTCCG*
Cfap97d1	*TCACAGGCAAAGCACAACCT*	*TTCTGCCAGTCCATCTCCGA*
Cfap97d2	*GGGACGCTGGAGTTTGTGAT*	*AGTGGTTGGGCATTCTGGAC*
Gapdh	*AGAACATCATCCCTGCATCC*	*CAGTGAGCTTCCCGTTCAG*
